# Human Blood Vessel Organoids Penetrate Human Cerebral Organoids and Form a Vessel-Like System

**DOI:** 10.3390/cells10082036

**Published:** 2021-08-09

**Authors:** Yujin Ahn, Ju-Hyun An, Hae-Jun Yang, Dong Gil Lee, Jieun Kim, Hyebin Koh, Young-Ho Park, Bong-Seok Song, Bo-Woong Sim, Hong J. Lee, Jong-Hee Lee, Sun-Uk Kim

**Affiliations:** 1 Futuristic Animal Resource and Research Center (FARRC), Korea Research Institute of Bioscience and Biotechnology (KRIBB), Ochang 28116, Korea; ayj0813@kribb.re.kr (Y.A.); ajh91@kribb.re.kr (J.-H.A.); baboi87@kribb.re.kr (H.-J.Y.); leedg@kribb.re.kr (D.G.L.); jieun622@kribb.re.kr (J.K.); hyebin627@kribb.re.kr (H.K.); pyh2877@kribb.re.kr (Y.-H.P.); sbs6401@kribb.re.kr (B.-S.S.); embryont@kribb.re.kr (B.-W.S.); 2 Department of Functional Genomics, KRIBB School of Bioscience, Korea University of Science and Technology, Daejeon 34113, Korea; 3 Department of Medical Life Sciences, College of Medicine, The Catholic University of Korea, Seoul 06591, Korea; 4 College of Medicine and Medical Research Institute, Chungbuk National University, Cheongju 28644, Korea; leehj71@gmail.com; 5 Research Institute, eBiogen Inc., Seoul 04785, Korea; 6 National Primate Research Center (NPRC), Korea Research Institute of Bioscience and Biotechnology (KRIBB), Ochang 28116, Korea

**Keywords:** vascularization, blood vessel organoid, cerebral organoid, blood-brain barrier, vessel structure, organoids, blood vessel formation, brain organoid

## Abstract

Vascularization of tissues, organoids and organ-on-chip models has been attempted using endothelial cells. However, the cultured endothelial cells lack the capacity to interact with other somatic cell types, which is distinct from developing vascular cells in vivo. Recently, it was demonstrated that blood vessel organoids (BVOs) recreate the structure and functions of developing human blood vessels. However, the tissue-specific adaptability of BVOs had not been assessed in somatic tissues. Herein, we investigated whether BVOs infiltrate human cerebral organoids and form a blood–brain barrier. As a result, vascular cells arising from BVOs penetrated the cerebral organoids and developed a vessel-like architecture composed of CD31^+^ endothelial tubes coated with SMA^+^ or PDGFR^+^ mural cells. Molecular markers of the blood-brain barrier were detected in the vascularized cerebral organoids. We revealed that BVOs can form neural-specific blood-vessel networks that can be maintained for over 50 days.

## 1. Introduction

As two-dimensional culture systems are unsuitable for sustaining the structures and functions of human tissues, and as most human tissues are difficult to biopsy and their use is tightly regulated by ethical regulations, organoids have been actively developed and applied to drug discovery and regenerative medicine over the last decade [1]. Although animal models have been used as substitutes for human tissues, interspecies differences in cell types, cell structures, and drug sensitivities restrict their applicability to humans.

Vascularization of organoids and organ-on-chip technology is being actively investigated to determine how vascular cells affect the differentiation or quiescence of other somatic cells and provide oxygen and nutrients to non-vascular cells. In previous studies, vascular structures were established in cortical organoids using cultured endothelial cells (ECs) [2,3]; however, cultured ECs lack tubulogenesis and tissue-specific adaptability, rendering them unlikely to develop blood vessels in vivo [4]. Also, endothelial tubes alone are insufficient to recreate the full vascular structure, consisting of ECs, smooth muscle cells (SMCs) or pericytes (PCs), astrocytes, and extracellular matrix [5].

In previous research, human blood vessel organoids (hBVOs) derived from human stem cells have been used to recreate human vascular structures and functions [6,7]. hBVOs are composed of endothelial networks coated with mural cells and enveloped in a basement membrane [6,7]. To date, the interactions between hBVOs and other cell types have not been investigated [6].

To investigate the interactions between blood vessels and other somatic cell types, we vascularized human cortical organoids (hCOs). As vascularization of the central nervous system (CNS) is initiated outside of the CNS [8], we attempted to sprout differentiated vessels within hCOs, rather than inducing the typical process of vasculogenesis.

In this study, BVOs were dissociated into clumps and co-cultured with cortical organoids to simulate embryonic neurovascular development. As a result, the hBVO clumps attached to each other and formed vessel-like structures containing tubes with an open lumen in the cortical organoids. We examined various vascular cell populations, blood–brain barrier (BBB) structures, interior cell death, and cortical cell characteristics in vascularized hCOs (vhCOs). The results indicated that hBVOs have vascularization capacity in cells of neural lineage. vhCOs may serve as a model to investigate human cortical development and neurovascular diseases and injuries. Furthermore, they can be used as a surrogate for drug screening and brain-vessel pharmacodynamics.

## 2. Materials and Methods

### 2.1. Human Induced Pluripotent Stem Cells (hiPSCs) and Culture Conditions

The hiPSCs were reprogrammed from primary human bone marrow cells (Allcells, Emeryville, CA, USA) as described previously [9]. hiPSCs were maintained in Matrigel (Corning, 354254)-coated plates containing TeSR E8 medium (Stemcell Technologies, Vancouver, BC, Canada) in a 37 °C incubator under 5% CO_2_. Cells were passaged every 5 days using the enzyme-free dissociation reagent ReLeSR (Stemcell Technologies, Vancouver, BC, Canada) under feeder-free conditions. hiPSCs were tested for mycoplasma contamination regularly and were used within 50 passages in this study. Cells were positive for the hiPSC pluripotency cell-surface markers SSEA3, SSEA4, and TRA-1-60, and for the transcription factors SOX2, Nanog, and OCT4, as determined by flow cytometry.

### 2.2. Construction of an Enhanced Green Fluorescent Protein (EGFP)-Overexpressing Cell Line

A cell line overexpressing the EGFP transgene was generated using clustered regularly interspaced short palindromic repeats (CRISPR)/Cas9 technology. First, to target exon 1 of beta 2-microglobulin, the guide RNA sequence 5′-CGGAGCGAGACATCT-3′ was ligated to the pCAG-SpCas9-GFP-U6-gRNA plasmid (Addgene plasmid #79145) and a donor template containing the EF1a-EGFP and G418-resistance expression cassette flanked by homology arms. Next, we transfected the CRISPR/Cas9 and donor template plasmids into hiPSCs using 4D Nucleofector (Lonza, Basel, Switzerland) following the manufacturer’s instructions. Briefly, the cells were dissociated using accutase (Stemcell Technologies, Vancouver, BC, Canada), after which 1 × 10^6^ cells were spun down at 600 rpm for 5 min, resuspended in 100 μL P3 Primary Cell Nucleofector^TM^ Solution (Lonza, Basel, Switzerland), mixed with 1.5 μg CRISPR/Cas9 plasmid and 1.5 μg donor template, transferred to a Nucleocuvette^TM^ vessel, and electroporated using program CM-130. The transfected cells were seeded in Matrigel (Corning, 354234)-coated 6-well plates containing E8 medium supplemented with 5 μm Y27632 (Tocris, Bristol, UK) and then selected using 500 μg/mL geneticin (Thermo Fisher Scientific, Waltham, MA, USA). Single-cell colonies were transferred to Matrigel-coated 24-well plates. Genotyping was performed by PCR and genomic DNA sequencing.

### 2.3. Generation of BVOs

hiPSCs were dissociated into single cells using accutase with a 70-μm strainer (Falcon), and 2000 cells were distributed per well in 96-well ultra-low attachment plates (Corning) in E8 medium containing 5 μm Y-27632. The plate was spun down at 1500 rpm for 5 min and then incubated for 24 h. Aggregated cells were treated with 12 μm CHIR99021 (Tocris, Bristol, UK) and 30 ng/mL BMP4 (Peprotech, Rocky Hill, NJ, USA) on days 0–2, and the medium was switched to that containing 100 ng/mL VEGF-A (Peprotech, Rocky Hill, NJ, USA) and 30 ng/mL forskolin (Stemgent) on days 3–5. On day 5, cell aggregates were embedded in a 1:2 Matrigel (Corning, 356231): collagen I (Advanced BioMatrix) solution and treated with StemPro34 (Gibco) containing 5% inactivated FBS (Thermo Fisher, Waltham, MA, USA), 100 ng/mL VEGF-A, and 100 ng/mL FGF-2 (R&D systems, Minneapolis, MN, USA). On day 7 or 8, ECs sprouted from the aggregates, and vascular networks were established. On day 10, individual BVOs were isolated from the gel and maintained in 96-well ultra-low attachment plates until day 15.

### 2.4. Generation of Cortical Organoids and Co-Culture with BVOs

A total of 5000 cell aggregates were generated from hiPSCs and incubated for 4 days in neural induction medium: DMEM-F12 (Gibco) supplemented with 20% KO-serum (Thermo Fisher, Waltham, MA, USA), 2 mm GlutaMax (Gibco), 1 mm MEM-NEAA (Thermo Fisher, Waltham, MA, USA), 55 nm 2-mercaptoethanol (Sigma, St. Louis, MO, USA), 2 μm dorsomorphin (Stemcell Technologies, Vancouver, BC, Canada), and 2 μm A-83 (Stemcell Technologies, Vancouver, BC, Canada). On day 4 of brain organoid development, day 15 BVOs were washed twice with KO-DMEM (Gibco) and dissociated with 1 mg/mL collagenase B (Roche, Basel, Switzerland) at 37 °C for 1 h. The dissociated BVO cells were co-cultured with brain organoids in a second neural induction medium: DMEM-F12 supplemented with 10% inactivated FBS, 1% N2 (Thermo Fisher, Waltham, MA, USA), 1% P/S (Thermo Fisher, Waltham, MA, USA), 1 mm MEM-NEAA, 2 mm GlutaMax, 1 μm CHIR99021, and 1 μm SB431542 (Cayman). On day 7, organoids were embedded in Matrigel (Corning, 354234) and maintained in stationary culture. On day 13, organoids were transferred to an orbital shaker in neural maturation medium: DMEM/F12 supplemented with 1% N2, 2% B27 with vitamin A, 2 mm GlutaMax, 1 mm MEM-NEAA, 1% P/S, 55 nm 2-mercaptoethanol, and 2.5 μg/mL insulin. The medium was changed every 2 days.

### 2.5. Immunofluorescence Staining

Organoids were rinsed twice with PBS and incubated in cell recovery solution (Corning, 354253) at 4 °C for 1 h to remove Matrigel from hCOs, as it causes a non-specific signal. After rinsing twice with PBS, organoids were fixed in 4% paraformaldehyde at room temperature (RT) for 1 h and washed with PBS for at least 30 min. Organoids were dehydrated in 30% sucrose at 4 °C overnight after embedding in OCT compound (Sakura) at −80 °C. These frozen blocks were sectioned using a cryostat (Leica) to 30-μm thickness. Tissues were permeabilized and blocked in a solution filtered by 0.2 μm syringe filter (Sartorius, Gottingen, Germany): PBS with 3% FBS (Welgene, Taipei, Taiwan), 1% bovine serum albumin (MPBIO), 0.5% Triton X-100 (Sigma, St. Louis, MO, USA), and 0.5% Tween 20 (Sigma, St. Louis, MO, USA) for 1 h at RT with agitation. Primary antibodies were diluted in blocking buffer and incubated overnight at cold room. The information of used antibodies and dilution factor is attached to Supplementary Table S1. After three washes in PBS with TritonX-100 (PBST), samples were incubated with secondary antibodies in blocking buffer for 3 h at RT. After further washing in PBST, samples were counterstained with DAPI (4′,6-diamidino-2-phenylindole) and imaged using a Zeiss LSM700 microscope.

### 2.6. Dextran Permeability Assay

Texas Red dextran (Thermo Fisher Scientific, D1830, 70,000 MW, Waltham, MA, USA) was diluted to 200 μg/mL with culture medium [10,11]. Cultured organoids were transferred to a 48-well plate and the dextran-containing medium was added. The plate was incubated for 1 h at 37 °C in an incubator under 5% CO_2_ on a rocker (0.246× *g*). Organoids were then washed with PBS three times for 5 min each, transferred to an imaging plate (Perkin Elmer, #6055801, Waltham, MA, USA), and imaged under a confocal microscope, as described previously. Quantification of dextran intensity was conducted using ImageJ software. The mean fluorescence intensity at the core of each organoid was measured at depths of 0–60 μm.

### 2.7. Whole-Mount Immunostaining of Organoids

To identify the overall three-dimensional and interior morphologies of organoids, organoids were cleared using the CytoVista™ 3D culture clearing kit (Invitrogen, MAN0017942, Waltham, MA, USA) according to the manufacturer’s instructions. All procedures were conducted on a shaker. Matrigel removal and the fixation procedures are the same as those used for immunofluorescence staining. After at least 1 h of washing in PBS, organoids were immersed in a gradient series of methanol (50%, 80%, and 100%) at 4 °C for permeabilization. Then, the samples were washed at RT in a series of buffers: 20% dimethyl sulfoxide/methanol, 80% methanol, 50% methanol, PBS, and PBS with 2% TritonX-100. Samples were incubated in antibody penetration buffer at RT for 1 h and blocked with blocking buffer at 37 °C. Primary antibodies were diluted in antibody buffer, and samples were incubated in antibody solution overnight at 37 °C. Organoids were washed in washing buffer five times for 10 min and incubated with a diluted solution of secondary antibody and DAPI overnight at 37 °C. Samples were then washed 10 times for 10 min in washing buffer. Samples were dehydrated in increasing concentrations of methanol (50%, 80%, and 100%) and incubated in CytoVista tissue clearing reagent overnight at 4 °C. Z-stack imaging was conducted using the Zeiss LSM700 confocal microscope.

### 2.8. Quantification of the Vascular Network

To measure degree of vascularization, organoids were immunostained by CD31 antibody and the images were analyzed using AngioTool software [12].

### 2.9. Quantitative Reverse-Transcription PCR (qRT-PCR)

Organoids were washed with PBS and frozen in liquid nitrogen. Total RNA was extracted using the RNeasy Mini Kit (Qiagen, Hilden, Germany) and reverse-transcribed into cDNA using a random primer and reverse transcriptase (Toyobo, Osaka, Japan). qPCR was conducted on a real-time PCR system (Thermo Fisher Scientific, Waltham, MA, USA), and relative mRNA quantification was performed using the 2^ΔΔCT^ method.

### 2.10. Flow Cytometric Analysis

Organoids were dissociated using collagenase B (Gibco) for 30–40 min, followed by pipetting and washing in Dulbecco’s PBS (Welgene, Taipei, Taiwan) containing 5% FBS. Single cells were stained with fluorescence-conjugated antibodies for 30 min at 4 °C. For staining of intracellular antigens, cells were fixed in 4% paraformaldehyde and permeabilized with 0.1% saponin (Sigma, St. Louis, MO, USA). Cells were stained with primary antibodies diluted in PBS containing 5% FBS. The information of used antibodies and dilution factor is attached to Supplementary Table S1. Data were collected on the FACS Aria flow cytometer (BD, Franklin Lakes, NJ, USA) and analyzed using FlowJo.

### 2.11. Statistical Analysis

Quantitative analysis of each dataset was performed using GraphPad Prism. All data are presented as means ± standard error of the mean.

## 3. Results

### 3.1. Generation of hBVOs

**Figure 1:**
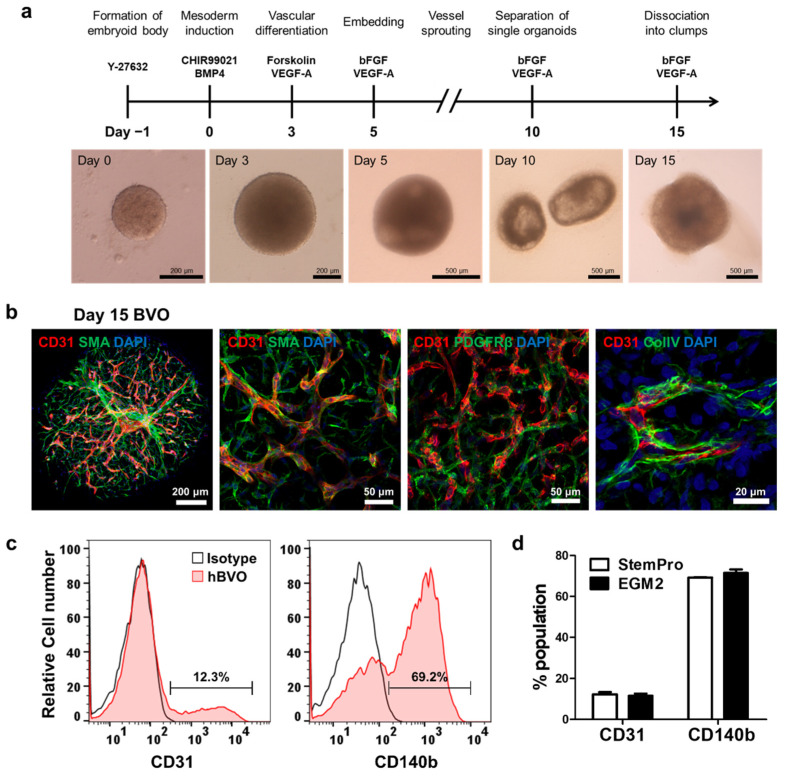
Generation of blood vessel organoids. (**a**) Schematic diagram of the protocol. (**b**) Endothelial networks showing the establishment of blood vessel organoids and vascular networks coated with pericytes (PDGFRβ^+^), smooth muscle cells (SMA^+^), and basement membrane (collagen type IV [ColIV]^+^). (**c**), Representative FACS histogram showing the percentages of endothelial cells (CD31^+^) and pericytes (CD140b^+^) in the blood vessel organoids (*n* = 9). (**d**) Histogram showing no significant differences in the cell population (endothelial cells and pericytes) of hBVOs between culture media (StemPro-34 and EGM2). Bars and error bars represent the mean ± SD of results in triplicate experiments (*n* = 9); unpaired, two-tailed student’s *t*-test.

hBVOs were derived from hiPSCs via a series of differentiation protocols (Figure 1a). hiPSC aggregates were induced to form mesoderm via treatment with CHIR99021 and BMP4 and subsequently differentiated into vascular cells via treatment with VEGF-A and forskolin. The organoids were embedded in collagen I/Matrigel solution containing VEGF-A and bFGF on day 5. After the vessels sprouted, BVOs were separated into individual organoids on day 10. Angiogenic ECs were assembled into hBVOs after 5 days of growth. Confocal imaging showed mesh-like CD31^+^ endothelial networks wrapped in SMA^+^ SMCs or PDGFRβ^+^ PCs and coated with collagen IV^+^ basement membrane in the hBVOs on day 15 (Figure 1b). Thus, hBVOs were rapidly generated from hiPSCs within 2 weeks and were composed of the cell types and extracellular matrices typical of in vivo blood vessels, and not simply endothelial tubes.

In the human CNS, relative to other tissues, blood vessels in the brain show an extraordinarily high ratio of PCs to ECs (1:1), which sustains the integrity of the BBB [13]. Flow cytometry was employed to measure the EC:PC ratio of hBVOs. The hBVOs consisted of 12.3 ± 1.8% ECs and 69.2 ± 0.38% PCs (Figure 1c), indicating an approximately five-fold greater density of PCs than ECs. To increase the proportion of ECs, we attempted to optimize culture media by switching StemPro34-based maturation medium to EGM-2, which is optimized for the culture of primary ECs. Although the maturation medium was changed, the cell population ratio did not significantly differ between the two conditions (Figure 1d). In this study, hBVOs were maintained by StemPro34-based medium.

### 3.2. Vascularization of Cerebral Organoids

**Figure 2:**
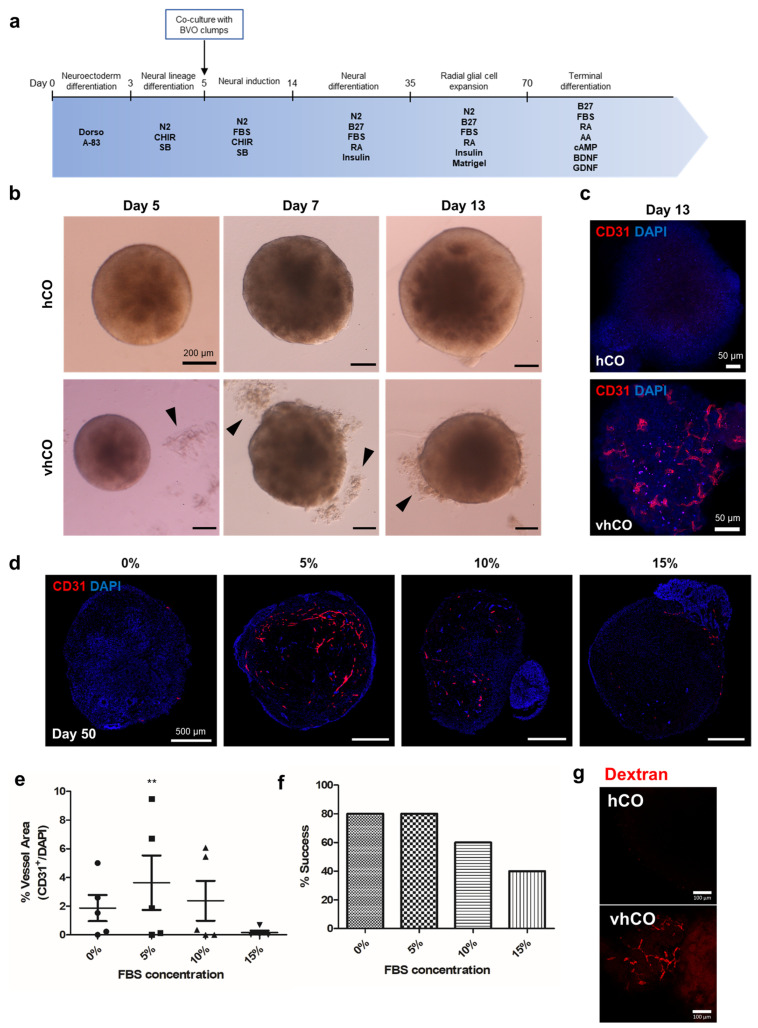
Generation of vascularized cerebral organoids. (**a**) Schematic diagram of the procedure used to generate vascularized cerebral organoids. (**b**) Blood vessel clumps (black arrowheads) fused to neural organoids generated on day 5 in the co-culture of developing cerebral organoids. (**c**) CD31^+^ endothelial cells were observed on day 13 of vhCO development. (**d**) CD31^+^ endothelial networks generated under different FBS conditions. (**f**) The percentage of organoids succeed to generate vessels after co-culture in different FBS concentrations. (**e**) Quantification of vessels performed using AngioTool. Data are plotted as means ± standard deviation. ** *p* < 0.01; one-way ANOVA. (**g**) Dextran was perfused on vhCO. (**f**) Ratios of successful vascularization under various FBS concentrations.

To examine whether the hBVOs can vascularize cerebral organoids, vascular plexuses were isolated from hBVOs and attached to hCOs on day 5, which is assumed to be before initiation of neural cell differentiation. To determine the number of cells comprising hCOs on day 5, hCOs were dissociated by accutase and counted using hemocytometer. As a result, hCOs consisted of approximately 2.16 × 105 cells per organoid (*n* = 4) and the number of cells comprising single BVO was determined in the same way. Based on these calculations, BVOs on day 15 were dissociated into clumps, the appropriate number of clumps were added to hCOs at the ratio of 1:10 for BVO:hCO with medium, and maintained (Figure 2a). The plexus fragments attached to hCOs were then embedded in diluted Matrigel containing neural induction medium (3:2 ratio) (Figure 2b). After stationary culturing until day 13, organoids were transferred to a shaker and further cultured with agitation. CD31^+^ vessel-like cells were detected on the surface of organoids on day 13 (Figure 2c). Co-culturing of hBVOs and hCOs produced vhCOs.

Next, we optimized the FBS concentration in the culture medium. Medium containing 0%, 5%, 10%, or 15% FBS was administered, and vhCOs in the 5% FBS medium showed significantly enhanced vascular-like network formation compared with the other FBS concentrations (Figure 2d,e). Under the 0% and 5% FBS conditions, 80% of embedded vhCOs successfully formed CD31^+^ endothelial-like networks on day 50 (Figure 2f). Thus, 5% FBS supplementation was optimal for vascularization of hCOs. To test the functionality of vascular structure, we performed dextran perfusion assay, which is commonly used to visualize blood vessels. After an hour of incubation in 70 kDa Texas-Red dextran containing media, the tubular structure in vhCOs was brightly visualized while hCOs showed no fluorescence signal (Figure 2g). This result directly evidenced the existence of perfusable vascular networks in vhCOs not in hCOs. However, this does not mean that the vascular-like structures in vhCOs have BBB-like permeability.

### 3.3. Identification of vhCOs

**Figure 3:**
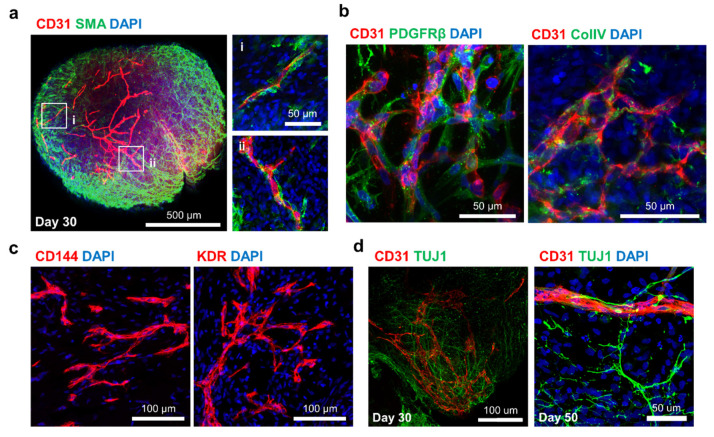
Identification of vessel-like structures in the vascularized cerebral organoids. (**a**) CD31^+^ endothelial networks of the vhCOs coated with SMA+ smooth muscle cells, similar to typical blood vessels. (**b**) Mesh-like endothelial plexuses surrounded by PDGFR+ pericytes and a collagen IV (ColIV)+ basement membrane. (**c**) Formation of vessel-like structures from endothelial progenitor cells (CD144^+^ and KDR^+^). (**d**) Coexistence of TUJ1^+^ neural networks and CD31^+^ vascular networks maintained for up to 50 days.

To determine whether the vessel-like tubes in vhCOs exhibit the typical structure of blood vessels, immunofluorescence staining was performed on day 30. The endothelial-like tubes were coated with abundant SMA^+^ SMC-like cells (Figure 3a). Moreover, PDGFRβ^+^ PCs and collagen IV^+^ basement membranes were detected on the periphery of endothelial-like tubes (Figure 3b). CD144^+^ or KDR^+^ endothelial progenitor-like cells were also present in the vessel-like structures of vhCOs (Figure 3c). Confocal imaging of vhCOs showed a complex network consisting of neural cells and vascular-like structures, which was evidenced by detection of TUJ1^+^ neuronal cells and CD31^+^ ECs on day 30 and day 50 (Figure 3d). These results suggested that a typical vascular-like structure formed in the vhCOs and coexisted with neural-like networks up to day 50.

**Figure 4:**
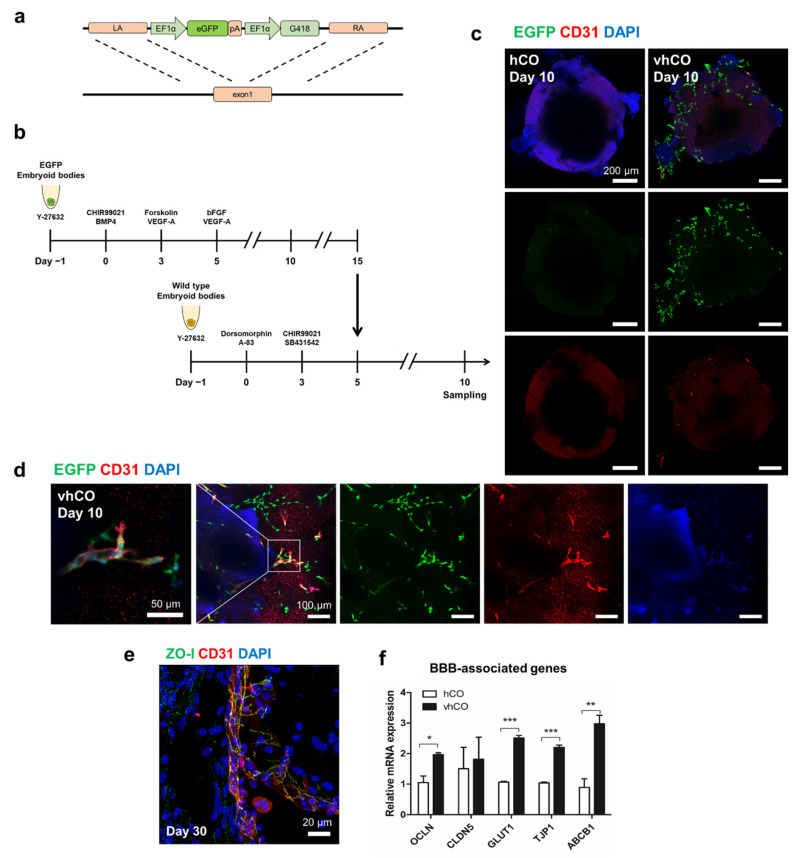
Characterization of blood-brain barrier-like structures in vhCOs. (**a**) The EGFP transgene template used to generate cells overexpressing EGFP. (**b**) EGFP-expressing blood vessel organoids co-cultured with wild-type cerebral organoids. (**c**) A few EGFP^+^ cells expressed the CD31^+^ endothelial marker in the vhCOs. (**d**) In magnificent images, CD31-expressing cells co-localized with EGFP^+^ cells. (**e**) ZO-I^+^ tight junction proteins detected between endothelial cells. (**f**) Expression of blood–brain barrier markers (occludin, claudin-5, glucose transporter1, ABCB1 and tight junction protein1) detected by RT-qPCR. Day 15 BVOs, day 18 hCOs, and day 18 vhCOs were analyzed (*n* = 8). * *p* < 0.05, ** *p* < 0.01, *** *p* < 0.001; unpaired, two-tailed student’s *t*-test. (**g**) GFAP^+^ astrocyte-like cells were non-covered CD31^+^ endothelial tubes.

To determine whether these ECs originated from BVOs or differentiated from hCOs, we differentiated EGFP-expressing iPSCs into BVOs (Figure 4a,b). Confocal images taken after 3 days of co-culture showed EGFP^+^ cells exist on the surface of vhCOs, but not on that of hCOs (Figure 4c). Consistent with the data shown in Figure 1c, a part of EGFP^+^ cells expressed CD31, a common EC marker. Meanwhile, almost all the CD31^+^ ECs expressed EGFP (Figure 4d). This result shows that vessel networks in vhCOs originated from hBVOs.

The human brain vasculature forms a highly selective barrier containing multiple cell types, known as the BBB [14]. Specifically, the tight junctions between ECs strictly regulate molecular entry from the bloodstream [15]. To investigate the BBB characteristics of the vessel-like structures, tight junction (ZO-I) and astrocyte (GFAP) markers were detected in vhCOs by immunofluorescence staining. ZO-I^+^ tight junction proteins were located between ECs in vhCOs on day 30 (Figure 4e). Also, RT-qPCR revealed that the expression levels of tight-junction-related genes (Occludin, glucose transporter1, ABCB1, and tight junction protein1) were significantly upregulated in vhCOs compared to hCOs (Figure 4f). However, the GFAP^+^ astrocyte-like cells did not cover CD31^+^ endothelial tubes in vhCOs like in vivo neurovasculature (Figure 4g). Although the vhCOs expressed tight junctions proteins related to BBB, it did not show similar in vivo-like morphological phenotypes.

## 4. Discussion

Functional vasculature in organoids is essential for closely recreating primary tissues. Vascularized organoids would be valuable for clinical research on various organotypic disease models, especially for neurovascular diseases such as stroke, atherosclerosis, aneurysm, and moyamoya disease, because brain vessels are difficult to acquire from patients, and rodent brain cytoarchitectures do not resemble that of the human brain.

Moreover, blood vessels are not generated de novo from the human neural tube [8,16]. At 6 weeks post-conception in human embryos, NPCs express VEGF, resulting in an elevated VEGF concentration inside the ventricle relative to outside of the ventricle [17]. Because the direction of vascular infiltration is determined by this VEGF gradient, vascular cells in ventral arterial tracts and cardinal veins sprout toward the ventricle [16,17]. Therefore, the procedure of neurovascular development is similar to that of generating vessels in hCOs by contact with an artificial vascular plexus. In our results, hCOs were vascularized from BVO clumps, and vessel-like structures were maintained for over 50 days. The mural cells are embedded in extracellular matrix such as a basement membrane and surrounded on the endothelial tubes. Also, astrocytes are close to the vascular-like structure. It resembles the cytoarchitecture of a neurovascular unit in the human cortex, not simply endothelial tubes [18,19].

In the brain, vascular cells promote NSC quiescence or self-renewal and differentiation via cell–cell contact signaling or secretion of angiocrine factors [20,21,22,23,24]. As a result, a vascular system, in addition to nutrients and oxygen, is crucial for the patterning of neurons. A recent study reported that human brain organoids develop more rapidly than human primary tissues [25]. The absence of vascular cells is assumed to affect the developmental rate, as they induce the quiescence, proliferation, differentiation, and maturation of NPCs [26]. Therefore, vascularization of hCOs may slow their developmental rate, preventing premature development and thus mimicking primary tissues better than ordinary hCOs.

In conclusion, incorporation of BVOs into brain organoids is a robust method for generating vascularized human brain organoids. The endothelial tubes are coated with mural cells and enveloped in a basement membrane, similar to typical blood vessels. They are well distributed on the cerebral organoids. Although cell–cell communication and its outcome require further investigation, vascularized cerebral organoids may offer an affordable and efficient platform for producing neurovascular disease models and thereby facilitating elucidation of the pathological mechanisms.

## Data Availability

All data that support the finding of this study are available upon reasonable request from the corresponding author.

## Supplementary Materials

The following are available online at https://www.mdpi.com/article/10.3390/cells10082036/s1, Figure S1: Confocal image of GFAP^+^ astrocytes in the vhCO, Table S1: Antibodies, Table S2: Primer sequence for qRT-PCR.
