# Supplementary material for: Human Blood Vessel Organoids Penetrate Human Cerebral Organoids and Form a Vessel-Like System
Source: Cells. 2021 Aug 9;10(8):2036. doi: 10.3390/cells10082036 (PMC8393185; doi:10.3390/cells10082036)
Supplement: Supplementary file 1 [file cells-10-02036-s001.zip › cells-1313585-supplementary.pdf]

Table S1. Antibodies

Primary antibodies	Target	Supplier	Catalog no.	Host species	Dilution factor	Experiment
CD31	Endothelial cells	BD Pharmingen™	555445	Mouse	1:100	FACS
CD31	Endothelial cells	Dako	M082329-2	Mouse	1:300	IHC (Cryo/Whole)
CD31	Endothelial cells	Neuromics	403392	Goat	1:500	IHC (Cryo/Whole)
CD31	Endothelial cells	thermo	MA5-29474	Rabbit	1:200	IHC (Cryo)
CD144	Early endothelial cells	R&D systems	AF938-SP	Goat	1:100	IHC (Whole)
KDR	Early endothelial cells	abcam	ab75769	Rabbit	1:250	IHC (Whole)
PDGFRβ	Pericytes	BD Pharmingen™	558821	Mouse	1:100	FACS
PDGFRβ	Pericytes	R&D systems	AF385-SP	Goat	1:400	IHC (Whole)
SOX2	Neural stem cells	Abcam	ab97959	Rabbit	1:500	IHC (Whole)
SOX2	Neural stem cells	Santa Cruz	sc-365823	Mouse	1:100	IHC (Whole)
TUJ1	Neurons	Biolegend	801201	Mouse	1:300	IHC (Whole)
GFAP	Astrocytes	Dako	Z0334	Rabbit	1:300	IHC (Cryo)
SMA	Smooth muscle cells	Sigma	a2547-100ul	Mouse	1:500	IHC (Whole)
Collagen type IV	Basement membrane	Millipore	AB769	Goat	1:400	IHC (Whole)
ZO-I	Tight junction proteins	Invitrogen	33-9100	Mouse	1:600	IHC (Cryo)
Tra-1-60	hiPSC stemness	BD Pharmingen	560173	mouse	1:100	FACS
SSEA4	hiPSC stemness	BD Biosciences	560308	Mouse	1:100	FACS
SSEA3	hiPSC stemness	BD Pharmingen	560237	rat	1:100	FACS
Sox2	hiPSC stemness	BD Biosciences	561593	Mouse	1:100	FACS
Oct3/4	hiPSC stemness	BD Pharmingen	560217	mouse	1:100	FACS
Nanog	hiPSC stemness	BD Pharmingen	560483	mouse	1:100	FACS

Secondary antibodies	Target	Supplier	Catalog no.	Host species	Dilution factor	Experiment
Alexa Fluor 488-anti-Goat IgG	Goat IgG	Thermo Fisher	A11055	Donkey	1:200	IHC (Cryo/Whole)
Alexa Fluor 555-anti-Goat IgG	Goat IgG	Thermo Fisher	A21432	Donkey	1:200	IHC (Cryo/Whole)
Alexa Fluor 488-anti-Rabbit IgG	Rabbit IgG	Thermo Fisher	A21206	Donkey	1:200	IHC (Cryo/Whole)
Alexa Fluor 555-anti-Rabbit IgG	Rabbit IgG	Thermo Fisher	A31572	Donkey	1:200	IHC (Cryo/Whole)
Alexa Fluor 488-anti-Mouse IgG	Mouse IgG	Thermo Fisher	A21202	Donkey	1:200	IHC (Cryo/Whole)
Alexa Fluor 555-anti-Mouse IgG	Mouse IgG	Thermo Fisher	A31570	Donkey	1:200	IHC (Cryo/Whole)

Table S2. Primer sequence for qRT-PCR

Primers	Tm (C°)	Sequence (5' to 3')	Fragment size (bp)	Reference PMID
GLUT1_F	58.9	CTGCTCATCAACCGCAAC	82, 129	23302780
GLUT1_R	59.4	CTTCTTCTCCCGCATCATCT	82, 129	23302780
ABCB1_F	61.4	CCCATCATTGCAATAGCAGG	157	25089713
ABCB1_R	55.6	GTTCAAACTTCTGCTCCTGA	157	25089713
CLDN5_F	63.6	CTCTGCTGGTTCGCCAACAT	75	30032046
CLDN5_R	60.3	CAGCTCGTACTTCTGCGACA	75	30032046
OCLN_F	59.7	ACAAGCGGTTTTATCCAGAGTC	89	30032046
OCLN_R	60.4	GTCATCCACAGGCGAAGTTAAT	89	30032046
TJP1-F	60	GGAGAGGTGTTCCGTGTTGT	253	25027596
TJP1-R	60	GAGCGGACAAATCCTCTCTG	253	25027596

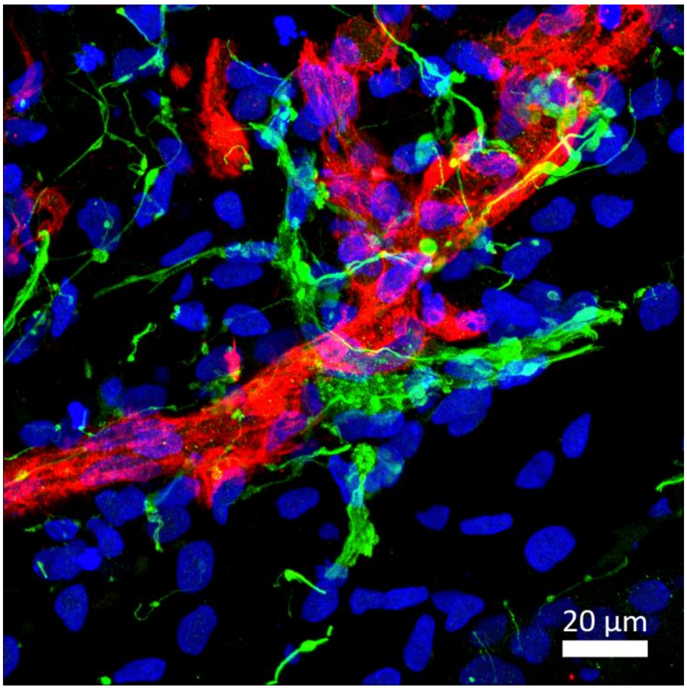

Figure S1. GFAP^{+} astrocyte-like cells were non-covered CD31^{+} endothelial tubes.
